# Angiotensin II Type 1 Receptor Antagonist Attenuates Lacrimal Gland, Lung, and Liver Fibrosis in a Murine Model of Chronic Graft-Versus-Host Disease

**DOI:** 10.1371/journal.pone.0064724

**Published:** 2013-06-06

**Authors:** Saori Yaguchi, Yoko Ogawa, Shigeto Shimmura, Tetsuya Kawakita, Shin Hatou, Shingo Satofuka, Shigeru Nakamura, Toshihiro Imada, Hideyuki Miyashita, Satoru Yoshida, Tomonori Yaguchi, Yoko Ozawa, Takehiko Mori, Shinichiro Okamoto, Yutaka Kawakami, Susumu Ishida, Kazuo Tsubota

**Affiliations:** 1 Department of Ophthalmology, Keio University School of Medicine, Tokyo, Japan; 2 Division of Cellular Signaling, Institute for Advanced Medical Research, Keio University School of Medicine, Tokyo, Japan; 3 Division of Hematology, Department of Internal Medicine, Keio University School of Medicine, Tokyo, Japan; 4 Department of Ophthalmology, Hokkaido University Graduate School of Medicine, Sapporo, Japan; National Taiwan University Hospital, Taiwan

## Abstract

Chronic graft-versus-host disease (cGVHD), a serious complication following allogeneic HSCT (hematopoietic stem cell transplantation), is characterized by systemic fibrosis. The tissue renin-angiotensin system (RAS) is involved in the fibrotic pathogenesis, and an angiotensin II type 1 receptor (AT1R) antagonist can attenuate fibrosis. Tissue RAS is present in the lacrimal gland, lung, and liver, and is known to be involved in the fibrotic pathogenesis of the lung and liver. This study aimed to determine whether RAS is involved in fibrotic pathogenesis in the lacrimal gland and to assess the effect of an AT1R antagonist on preventing lacrimal gland, lung, and liver fibrosis in cGVHD model mice. We used the B10.D2→BALB/c (H-2^d^) MHC-compatible, multiple minor histocompatibility antigen-mismatched model, which reflects clinical and pathological symptoms of human cGVHD. First, we examined the localization and expression of RAS components in the lacrimal glands using immunohistochemistry and quantitative real-time polymerase chain reaction (PCR). Next, we administered an AT1R antagonist (valsartan; 10 mg/kg) or angiotensin II type 2 receptor (AT2R) antagonist (PD123319; 10 mg/kg) intraperitoneally into cGVHD model mice and assessed the fibrotic change in the lacrimal gland, lung, and liver. We demonstrated that fibroblasts expressed angiotensin II, AT1R, and AT2R, and that the mRNA expression of angiotensinogen was greater in the lacrimal glands of cGVHD model mice than in controls generated by syngeneic-HSCT. The inhibition experiment revealed that fibrosis of the lacrimal gland, lung, and liver was suppressed in mice treated with the AT1R antagonist, but not the AT2R antagonist. We conclude that RAS is involved in fibrotic pathogenesis in the lacrimal gland and that AT1R antagonist has a therapeutic effect on lacrimal gland, lung, and liver fibrosis in cGVHD model mice. Our findings point to AT1R antagonist as a possible target for therapeutic intervention in cGVHD.

## Introduction

Chronic graft-versus-host disease (cGVHD), a multisystem chronic allo-immune and auto-immune disorder, is a serious and potentially life-threatening long-term complication of allogeneic HSCT (hematopoietic stem cell transplantation) [1].

Clinical manifestations of cGVHD include inflammation and fibrosis [2].

We previously reported that cGVHD is frequently related to dry eye, with 50% of patients developing or experiencing worsened pre-existing dry eye after HSCT [3], and that the lacrimal glands of cGVHD patients show marked fibrosis and inflammatory cell infiltration around medium-sized ducts in the interlobular areas [4]. Chronic pulmonary dysfunction occurs in 20% to 50% of cGVHD patients, depending on the donor source and the time interval after HSCT. The chronic lung injury is subdivided into two types: obstructive lung disease (OLD) and restrictive lung disease (RLD). In each type, collagen deposition and fibrosis development are observed either in the peribronchiolar space (OLD) or interstitial space (RLD) [5]. Evidence of cholestasis is present in approximately 80% of patients with cGVHD. In the histopathology of liver cGVHD, ductopenia, portal fibrosis, and chronic cholestasis reflect chronicity [6]. However, the pathophysiology of cGVHD is not completely understood, and an effective therapy for it has not been established.

Only a few animal models have been developed to examine cGVHD. Among them, the B10.D2→BALB/c (H-2^d^) MHC-compatible, multiple minor histocompatibility antigen-mismatched mouse shows several characteristics resembling human cGVHD. Its features include skin fibrosis with increased collagen deposition [7], [8], pulmonary fibrosis [7], inflammation and destruction of the salivary and lacrimal glands [9], and hepatic disease characterized by intrahepatic and extrahepatic bile duct mononuclear cell infiltration, followed by fibrous thickening and sclerosis of the bile duct wall [10], [11].

The systemic renin-angiotensin system (RAS) plays an important role in the endocrine regulation of blood pressure and of salt and water balance [12].

Angiotensinogen, the precursor of angiotensin, is secreted into the circulation and cleaved by renin to the decapeptide angiotensin I. Angiotensin I is subsequently converted to the octapeptide angiotensin II by angiotensin-converting enzyme (ACE) [13]. The action of angiotensin II, the major bioactive product of the RAS, is mediated mainly through its interaction with two pharmacologically defined receptor subtypes, type 1 (AT1R) and type 2 (AT2R), which are distributed in numerous tissues [14].

Besides the systemic RAS, many peripheral tissues are capable of generating RAS components. This so-called tissue RAS has various roles, including the promotion of inflammation and fibrosis [15]. The majority of these pathophysiological functions of angiotensin II are mediated through AT1R. Tissue RAS is present in the lacrimal gland [16], lung [17], and liver [18]. Angiotensin II is implicated in fibrotic pathogenesis in the lung and liver [17], [19]. Recent studies have shown that treatment with blockers of angiotensin II action, especially AT1R antagonists, can decrease inflammation and fibrosis of the kidney, lung, and liver in animal models of fibrotic disease [20]–[22].

In this study, we investigated the involvement of tissue RAS in fibrotic pathogenesis in the lacrimal gland and assessed the effect of an AT1R antagonist on the lacrimal gland, lung, and liver fibrosis in the B10.D2→BALB/c (H-2^d^) murine model of cGVHD.

## Materials and Methods

### B10.D2→BALB/c (H-2^d^) Murine Model of cGVHD

Eight-week-old male B10.D2 (H-2^d^, Sankyo Laboratory, Inc. Tokyo, Japan) and female BALB/c (H-2^d^) mice were utilized as BMT donors and recipients, respectively, to produce cGVHD, using a previously reported method [7], [8]. Briefly, female recipient mice were lethally irradiated with 700 cGy of X-ray (X-ray device; MRB-1520R-3; Hitachi Medical Co., Tokyo, Japan). Approximately 6 hours later, they were injected in the tail vein with male donor bone marrow (1×10^6^/mouse) and spleen cells (2×10^6^/mouse) suspended in RPMI medium 1640 (Invitrogen, Carlsbad, CA). A control group of female BALB/c recipient mice received male BALB/c spleen and bone marrow cells (syngeneic BMT, referred to as a control animals). To assess the engraftment efficiency, fluorescein in situ hybridization (FISH) for Y-chromosomes was performed using as peripheral blood cells, spleen cells from female recipient mice of male whole bone marrow transplantation (Materials and methods S1). After transplantation, the animals were kept under specific pathogen-free conditions in our animal facility at Keio University School of Medicine and supplied with autoclaved food and acidified water. In this study, mice were sacrificed via cervical dislocation 21 days after BMT. Day 21 was chosen because it is reported to be the earliest time point at which reliable changes in inflammation and fibrosis are observed, and it has been used for analyses in the same murine model of cGVHD [7], [8], [11].

All the animal experiments were conducted in accordance with the ARVO Statement for the Use of Animals in Ophthalmic and Vision Research. The ethics committee of the Keio University School of Medicine approved all the surgical interventions and animal care procedures. The protocol for this study was approved by the Ethics Committee on Animal Research of the Keio University School of Medicine (protocol # 09152).

### Effect of AT1R and AT2R Antagonists on Fibrosis

cGVHD model mice were treated with intraperitoneal injections of vehicle, (dimethylsulfoxide [DMSO] diluted with phosphate-buffered saline [PBS]), an AT1R antagonist (valsartan, LKT Laboratories, Inc. St. Paul, MN), and an AT2R antagonist (PD123319; Sigma, St. Louis, MO) every other day from day 1 to 21 after BMT. The groups were named the DMSO group, valsartan group, and PD123319 group. Valsartan was dissolved in DMSO, diluted with PBS, and intraperitoneally injected into mice at a dose of 10 mg/kg. The dilution rate of DMSO in PBS injected to the DMSO group was same as that of valsartan. PD123319 was dissolved in PBS and injected into mice at a dose of 10 mg/kg. The dose of 10 mg/kg for valsartan and PD123319 were used in other reports on tissue RAS [23]–[25]. To assess the hemodynamic effect of these compounds, we measured the systolic blood pressure (SBP), diastolic blood pressure (DBP), and heart rate (HR), using a computerized, noninvasive tail cuff system (MK-2000; Muromachi Kikai, Tokyo, Japan), before and after 1 week of daily injections of DMSO, valsartan, and PD123319. Neither valsartan nor PD123319 had any significant effect on the SBP, DBP, or HR after 1 week of daily treatments compared with baseline (data not shown). We also verified that the injected dose of DMSO, valsartan, and PD123319 did not cause any apparent morphological changes or inflammation in tissue sections of the three groups after 1 week of daily treatments (data not shown).

Mice were sacrificed via cervical dislocation 21 days after BMT.

### Collection of Murine Tissue

The mouse lacrimal glands were harvested for RNA extraction (snap-frozen in liquid nitrogen), fixed in 10% neutralized buffered formalin (Muto Pure Chemicals Co., Ltd. Tokyo, Japan), and embedded in paraffin for routine histological staining. The mouse lungs and livers were fixed in 10% neutralized buffered formalin (Muto Pure Chemicals Co., Ltd.), and embedded in paraffin for routine histological staining.

### Histopathologic Assessment of Tissue Specimens

Formalin fixed, paraffin-embedded tissue sections were processed using conventional histological techniques, including hematoxylin-eosin and Mallory staining [26], [27].

### Immunohistochemistry for CD45, HSP47, Ki-67, Collagen Type I, Collagen Type III, Angiotensin II, AT1R, and AT2R

Immunohistochemical experiments were performed on lacrimal gland samples. Paraffin-embedded sections were used for immunohistochemistry as previously described [28]. Briefly, consecutive 6-µm-thick paraffin-embedded sections were deparaffinized, rehydrated, and washed with PBS. The sections were then blocked with 10% goat serum (Invitrogen) for 30 minutes and then incubated overnight at 4°C with the following primary antibodies: rat anti-mouse CD45 (BD Biosciences, NJ), mouse anti-mouse heat shock protein 47 (HSP47) (StressGen Biotechnologies Corp., Victoria, BC, Canada), goat anti-mouse Ki-67 (Santa Cruz Biotechnology, Santa Cruz, CA), rat anti-mouse type I collagen (Chondrex, Inc., WA), rabbit anti-mouse collagen III (Abcam, MA), rabbit anti-mouse angiotensin II (Santa Cruz Biotechnology), rabbit anti-mouse AT1R (Santa Cruz Biotechnology) and rabbit anti-mouse AT2R (Santa Cruz Biotechnology).

After being washed with PBS, the sections were treated with a peroxidase-conjugated secondary antibody (Nichirei Bioscience Inc., Tokyo, Japan) for 45 minutes, and washed with PBS. The reaction products were developed with a mixture of 3,3′-diaminobenzine-4 HCI (DAB) and H2O2 (Muto Pure Chemicals Co., LTD.). Antigen unmasking was performed by autoclaving at 120°C for 20 minutes for HSP47 and Ki-67 or microwaving for 21 minutes for CD45, type I collagen, collagen III, and angiotensin II, AT1R, and AT2R, in 10 mM sodium citrate buffer. Isotype-matched corresponding IgG antibodies were used as negative controls.

CD45, a marker of cells of hematopoietic origin, except erythrocytes, was used to assess the degree of inflammation. HSP47, a collagen-specific molecular chaperon, was used as a specific marker for mouse fibroblasts [29]. The co-expression in interstitial fibroblasts of HSP47 and Ki-67, type I collagen, or collagen III was examined by fluorescent double staining with mouse anti-mouse HSP47 and an Alexa Fluor 568-conjugated goat anti-mouse IgG secondary antibody (Invitrogen/Molecular Probes), and goat anti-mouse Ki-67 with an Alexa Fluor 488-conjugated rabbit anti-goat IgG secondary antibody (Invitrogen/Molecular Probes), rat anti-mouse type I collagen with an Alexa Fluor 488-conjugated goat anti-rat IgG secondary antibody (Invitrogen/Molecular Probes), or rabbit anti-mouse collagen III with an Alexa Fluor 488-conjugated goat anti-rabbit IgG secondary antibody (Invitrogen/Molecular Probes). The co-expression in interstitial fibroblasts of HSP47 and angiotensin II, AT1R, and AT2R was examined by fluorescent double-staining with mouse anti-mouse HSP47 and an Alexa Fluor 568-conjugated goat anti-mouse IgG secondary antibody (Invitrogen/Molecular Probes), and rabbit anti-mouse angiotensin II, rabbit anti-mouse AT1R, or rabbit anti-mouse AT2R with an Alexa Fluor 488-conjugated goat anti-rabbit IgG secondary antibody (Invitrogen/Molecular Probes). Nuclei were counterstained with TO-PRO-3 (Invitrogen/Molecular Probes). The tissue sections used for fluorescent staining were mounted on glass slides and examined with a confocal microscope (LSM700-ZEN 2009, Carl Zeiss Microlmaging GmbH, Jena, Germany).

### Histological and Morphometric Analysis

Tissue sections of lacrimal gland stained for CD45 and HSP47 were assessed to quantify the CD45^+^ inflammatory cells and HSP47^+^ fibroblasts. Four randomly selected fields of interlobular areas, which contain medium-sized interlobular ducts, the reported main target of inflammation and fibrosis in the lacrimal gland of cGVHD patients [4], were captured at 200X magnification for each section using a Nikon Coolscope II (Nikon, Tokyo, Japan), and the number of CD45^+^ inflammatory cells and HSP47^+^ fibroblasts was counted. Tissue sections of lacrimal gland, lung, and liver subjected to Mallory staining were assessed for morphometric analysis. A minimum of eight randomly selected fields were captured at 200X magnification for each section using a Nikon Coolscope II (Nikon) and imported into a computerized image analysis system; Image J (NIH, Bethesda, MD). The degree of fibrosis 21 days after BMT was analyzed. For lacrimal gland tissue, the collagen deposition was quantified as the ratio of the blue-stained area to the total stained area and expressed as % fibrotic area [30]. For lung tissue, the interstitial space fibrosis was quantified as the ratio of the alveolar space to the total area. This method was previously reported to assess the degree of pulmonary fibrosis in the Scl GVHD murine model [7]. For liver tissue, the periductal fibrosis was quantified as the mean thickness of the blue-stained area around the intrahepatic bile duct. This method was previously reported to assess the degree of liver fibrosis in a murine model of cGVHD [11]. To assess the histological architecture and staining, each slide was reviewed twice, by each of three independent observers (S.Y., Y.O., and S.S.). Observers were blinded to which group the specimen was from in the morphometric observation. The sections selected for analysis were discussed, and finally, S.Y. analyzed the data.

### Quantitative Real-Time Polymerase Chain Reaction (PCR)

Total RNA was extracted from lacrimal glands using an RNeasy mini kit (Qiagen, Valencia, CA), and cDNA synthesis was performed using a Rever Tra Ace qPCR RT Kit (Toyobo Co., Ltd. Osaka, Japan). The primers for mRNA expression analysis by TaqMan real-time polymerase chain reaction (PCR) were purchased from Applied Biosystems (Applied Systems Inc, Streetsville, Ontario, Canada) for the housekeeping gene glyceraldehyde 3-phosphate dehydrogenase (GAPDH), HSP47, collagen type I alpha 1, collagen type I alpha 2, collagen type III alpha 1, angiotensinogen, prorenin, ACE, and AT2R. SYBER Green-based real-time PCR was performed for AT1R, sense 5′-TCACCTGCATCATCATCTGG-3′, antisense, -3′AGCTGGTAAGAATGATTAGG5′- primer was used as templates for AT1R amplification. Quantitative real-time PCR was performed using the Step One Plus system (Applied Biosystems). All data were analyzed with the 2ΔΔCT method, and the mRNA of GAPDH was used as the internal standard.

### Statistical Analyses

All results are expressed as the mean ± SD. The data were subjected to statistical analyses (unpaired Student's t-test and Bonferroni/Dunn test) to determine differences among the means of the experimental, treatment, and control groups. Differences were considered to be statistically significant at p<0.05.

## Results

### Histological Changes and Fibrotic Features of the Lacrimal Glands in cGVHD Model Mice 21 Days After BMT

We first confirmed that the engraftment efficiency in the cGVHD model mouse was high (88.8%), using FISH for Y-chromosomes (Figure S1). We then examined whether this cGVHD model would be useful for studying lacrimal gland inflammation and fibrosis. We characterized the inflammatory and fibrotic changes in this cGVHD mouse’s lacrimal gland. In all the experiments with this model, we compared the results with those from syngeneic BMT controls.

**Figure 1:**
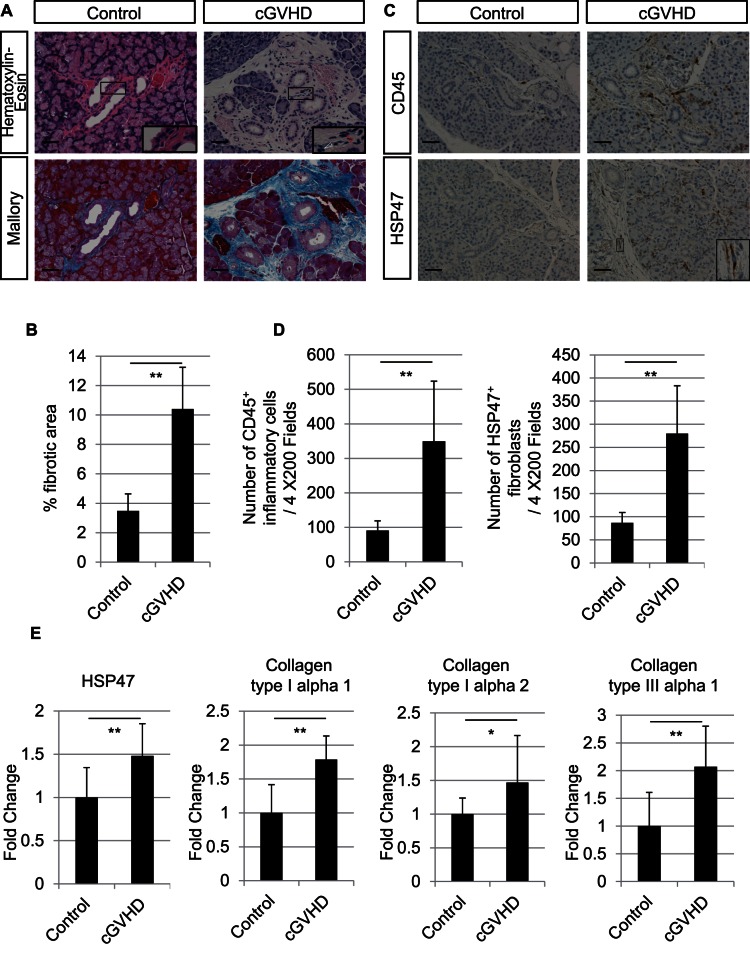
Fibrotic changes in control and cGVHD murine lacrimal glands. A: Light microscopy of cGVHD lacrimal glands showing the infiltration of inflammatory cells around medium-sized interlobular ducts, and a severely fibrotic interstitium that is more intense in the periductal areas. Inset: Infiltrated inflammatory cells (white arrowhead) and interstitial fibroblasts (red arrowhead). B: Collagen deposition was quantified as the ratio of the blue-stained area to the total stained area in Mallory staining and expressed as % fibrotic area. Collagen deposition was more severe in the cGVHD lacrimal glands compared with the controls (p = 0.000005). C: Immunostaining for CD45 and HSP47 in lacrimal glands shows intense brown staining of many cells in the interstitium and periductal areas in the cGVHD lacrimal glands. CD45-positive cells indicate infiltrating inflammatory cells. HSP47-positive cells are spindle-shaped cells with oval nuclei, which indicate fibroblasts (inset). D: The mean densities of CD45^+^ inflammatory cells and HSP47^+^ fibroblasts were significantly higher in the cGVHD compared with control lacrimal glands (CD45^+^ inflammatory cells; p = 0.001, HSP47^+^ fibroblasts; p = 0.0001). E: Quantitative real-time PCR revealed that the HSP47, collagen type I alpha 1, collagen type I alpha 2, and collagen type III alpha 1 expressions in the cGVHD group were higher than in the control group (HSP47; p = 0.004, collage type I alpha 1; p = 0.0002, collagen type I alpha 2; p = 0.03, collagen type III alpha 1; p = 0.002). Magnification, A: X200, C: X200. Scale bar, A: 50 µm, C: 50 µm. The results represent the mean ± SD. B: n = 9; D: n = 8; E: n = 9 in each group. E: The vertical axis shows the expression ratio of mRNAs. Fold change shows the expression ratio of mRNAs in the control and cGVHD group. The expression level in the control group was defined as 1. *p<0.05, **p<0.01 by Student's t-test.

In the lacrimal glands of the cGVHD model mice, hematoxylin-eosin staining showed inflammatory cell infiltration around the medium-sized interlobular ducts. Mallory staining showed a severely fibrotic interstitium in these periductal areas. These pathogenic fibrotic changes were more severe in the cGVHD compared with the control group (Figure 1A). We confirmed that the phenotype of the cGVHD mouse lacrimal gland closely resembles that of clinical samples of patients suffering from cGVHD [4]. The collagen deposition was also more severe in the cGVHD compared with the control lacrimal glands (p = 0.000005) (Figure 1B).

To assess the degree of inflammation and HSP47^+^ fibroblast involvement in the fibrosis of cGVHD model mice, we performed immunostaining for CD45 and HSP47. In the lacrimal glands of the control group, weak CD45 and HSP47 immunoreactivities were observed. In contrast, in the cGVHD lacrimal glands, we observed numerous CD45^+^ inflammatory cells and HSP47^+^ fibroblasts, mainly in the interstitium and periductal areas (Figure 1C).

The mean CD45^+^ inflammatory cell and HSP47^+^ fibroblast densities were significantly higher in the cGVHD than in the control group (CD45^+^ inflammatory cells; p = 0.001, HSP47^+^ fibroblasts; p = 0.0001) (Figure 1D). In addition, the mRNA expressions of HSP47, collagen type I alpha 1, collagen type I alpha 2, and collagen type III alpha 1, determined by quantitative real-time PCR analysis, were higher in the cGVHD than the control group (HSP47; p = 0.004, collage type I alpha 1; p = 0.0002, collagen type I alpha 2; p = 0.03, collagen type III alpha 1; p = 0.002) (Figure 1E). The difference between the effect of cGVHD on HSP47^+^ fibroblast density and the mRNA expression of HSP47 is owing to the difference in assessment area. The HSP47^+^ fibroblast density was measured in the interlobular areas, where the fibrosis was mainly observed, whereas the mRNA expression was measured using the whole lacrimal gland.

To confirm that the fibroblasts are activated, proliferate, and synthesize collagens in the fibrotic areas of cGVHD lacrimal glands, we performed immunofluorescence staining to examine the co-expression of HSP47 and Ki-67, collagen type I, or collagen type III. The fibroblasts in the cGVHD lacrimal glands co-expressed HSP47 and Ki-67, which were rarely detected in the control lacrimal glands (Figure S2). In the interlobular areas, the immunostainings of collagen type I and collagen type III were stronger, and more fibroblasts co-expressed HSP47 and collagen type I and collagen type III in the cGVHD than the control lacrimal glands, suggesting that fibroblasts in cGVHD lacrimal glands synthesize more collagens than do those in controls (Figure S2). These results show obvious lacrimal gland fibrosis in the cGVHD model mice, with the features of increased collagen deposition, inflammatory cell infiltration, high levels of CD45^+^ inflammatory cell and HSP47^+^ fibroblast densities, and increased mRNA expressions of HSP47, collagen type I alpha 1, collagen type I alpha 2, and collagen type III alpha 1.

### Ducts and Vessels Express AT1R and AT2R, Interstitial Fibroblasts Express Angiotensin II, AT1R, and AT2R, and the mRNA of Angiotensinogen is Increased in the Lacrimal Glands of cGVHD Model Mice

**Figure 2:**
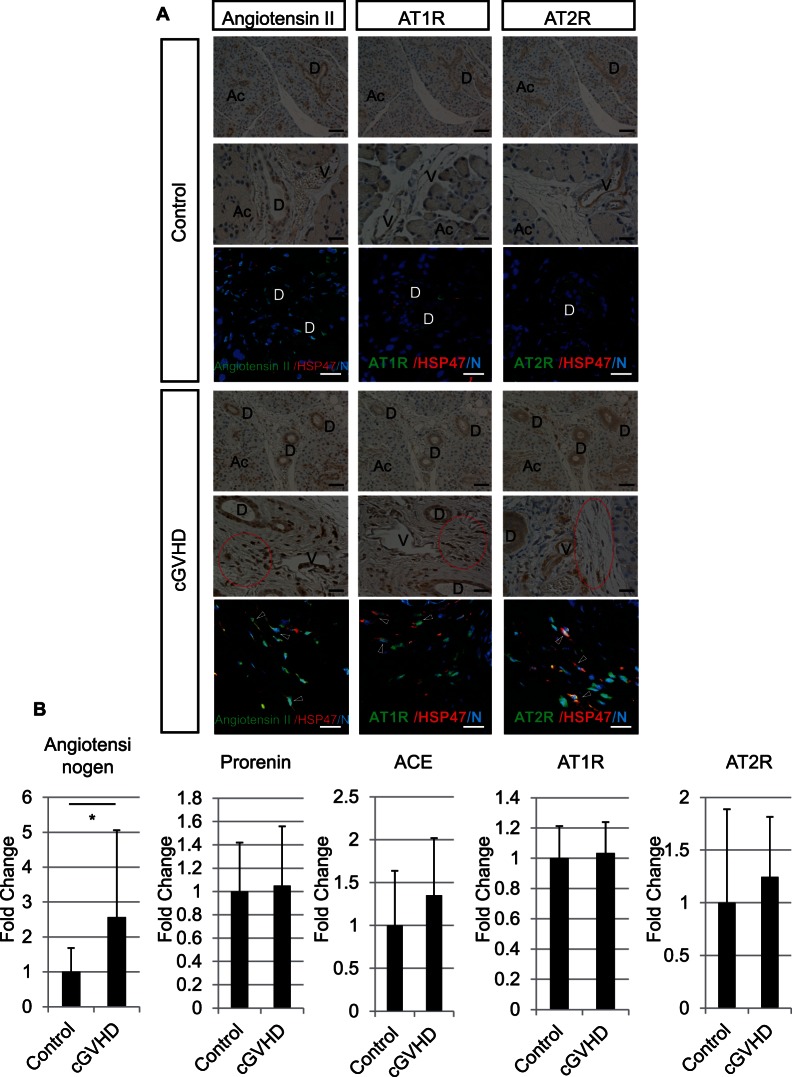
RAS components in control and cGVHD murine lacrimal glands. A: Immunohistochemical analysis showing that angiotensin II, AT1R, and AT2R were localized to the ducts and interstitial cells. There appeared to be markedly more positive-stained interstitial cells (circles), with stronger staining, in the cGVHD than in the control lacrimal glands. AT1R and AT2R were also localized to blood vessels. In immunofluorescence double-staining experiments, there appeared to be more interstitial fibroblasts co-expressing HSP47 and angiotensin II, AT1R, or AT2R (arrowhead) in the cGVHD than in the control lacrimal glands. B: Quantitative real-time PCR revealed that the angiotensinogen expression in cGVHD lacrimal glands was higher than that in controls (Angiotensinogen; p = 0.02, Prorenin; p = 0.86, ACE; p = 0.27, AT1R; p = 0.73, AT2R; p = 0.5). D, Duct; Ac, Acinus; V, Blood vessel. Magnification, A: X200 (Upper DAB staining panels), X400 (lower DAB staining panels and fluorescence staining). Scale bar, A: 50 µm (upper DAB staining panels), 25 µm (lower DAB staining panels and fluorescence staining). The results represent the mean ± SD. B: n = 9 in each group except for angiotensinogen (n = 16). B: The vertical axis shows the expression ratio of mRNAs. Fold change shows the expression ratio of mRNAs in the control and cGVHD group. The expression level in the control group was defined as 1. *p<0.05 by Student's t-test.

We next examined the localization of angiotensin II, AT1R, and AT2R by immunohistochemistry using specific antibodies. Angiotensin II, AT1R, and AT2R appeared to be localized exclusively to the ducts and interstitial cells in both the control and cGVHD lacrimal glands. In the ducts, angiotensin II was localized to the epithelial cells, AT1R to the basolateral membrane, and AT2R to the apical membrane. AT1R and AT2R were also localized to the blood vessels in both groups. These immunoreactive patterns of angiotensin II, AT1R, and AT2R were same as in the lacrimal glands from wild-type mice [16]. Markedly more interstitial cells appeared to be positive for angiotensin II, AT1R, and AT2R, with stronger staining, in the cGVHD samples than in the control. Some of the interstitial cells had a spindle-shaped morphology with oval nuclei, suggesting that they were fibroblasts. RAS components are also reported to be present in lacrimal gland fibroblasts from wild-type mice [16]. To confirm that the cells positive for angiotensin II, AT1R, and AT2R in the lacrimal glands were fibroblasts, we examined the co-expression of HSP47 and angiotensin II, AT1R, or AT2R in tissue sections. There appeared to be more interstitial fibroblasts co-expressing HSP47 and angiotensin II, AT1R, or AT2R in the cGVHD than in the control lacrimal glands (Figure 2A).

Next, we quantified the mRNA expression of RAS components (angiotensinogen, prorenin, ACE, AT1R, and AT2R) in the whole lacrimal glands by quantitative real-time PCR. Among the RAS components, the expression of angiotensinogen, the precursor of angiotensin II, was significantly higher in the cGVHD than in the control lacrimal glands (p = 0.02) (Figure 2B).

The interstitial fibroblasts were strongly positive for angiotensin II, AT1R, and AT2R. The mRNA expression of angiotensinogen was higher in the cGVHD than in the control lacrimal glands. No significant difference was observed in the mRNA expression of AT1R or AT2R in the lacrimal gland. It is likely that the increased AT1R and AT2R expressions in the fibroblasts were not apparent in the analysis using the whole lacrimal gland, because the fibrotic area where activated fibroblasts accumulate was limited to the interlobular periductal area in cGVHD, and the ducts and vessels also expressed AT1R and AT2R in both the control and cGVHD groups. Nevertheless, these results indicate that RAS may be activated in the fibrotic area and that the interstitial fibroblasts play a pivotal role in the cGVHD lacrimal gland.

### Lacrimal Gland Fibrosis in the cGVHD Model Mice is Inhibited by an AT1R Antagonist, but not by an AT2R Antagonist

We further analyzed whether AT1R and AT2R antagonists could prevent the development of lacrimal gland fibrosis in the cGVHD model mice.

**Figure 3:**
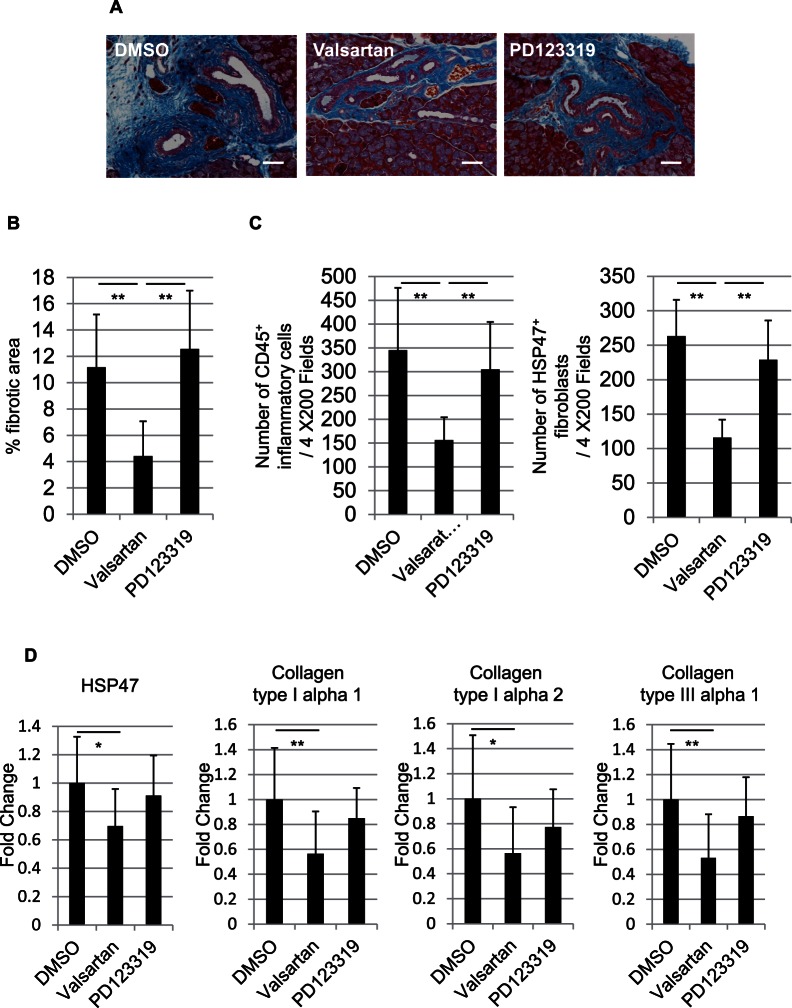
Fibrotic changes in the murine lacrimal glands of cGVHD mice treated with DMSO, valsartan, and PD123319. A: Mallory staining of the DMSO, valsartan, and PD23319 lacrimal glands showing that the fibrosis around the medium-sized interlobular ducts was decreased in the valsartan group. B: Collagen deposition was mildest in the valsartan group (p<0.01). C: The mean densities of CD45^+^ inflammatory cells and HSP47^+^ fibroblasts in the lacrimal glands were decreased in the valsartan group compared with the DMSO and PD123319 groups (both CD45^+^ inflammatory cells and HSP47^+^ fibroblasts: p<0.01). D: Quantitative real-time PCR revealed that the HSP47, collagen type I alpha 1, collagen type I alpha 2, and collagen type III alpha 1 expression in the valsartan group were significantly lower than those in the DMSO group (HSP47; p<0.05, collage type I alpha 1; p<0.01, collagen type I alpha 2; p<0.05, collagen type III alpha 1; p<0.01). Magnification, A: X200. Scale bar, A: 50 µm. The results represent the mean ± SD. B, C, D: DMSO n = 6, valsartan n = 8, PD123319 n = 8. D: The vertical axis shows the expression ratio of mRNAs. Fold change shows the expression ratio of mRNAs in the DMSO, valsartan, and PD123319 group. The expression level in the DMSO group was defined as 1. *p<0.05, **p<0.01 by Bonferroni/Dunn test.

Mallory staining of the lacrimal glands of cGVHD mice treated with DMSO, the AT1R antagonist valsartan, and the AT2R antagonist PD123319 revealed that the periductal fibrosis was decreased in the valsartan group compared with the DMSO or PD123319 group (Figure 3A). Similarly, collagen deposition was mildest in the valsartan group (p<0.01) (Figure 3B). The mean CD45^+^ inflammatory cell and HSP47^+^ fibroblast densities were significantly decreased in the valsartan group compared with the DMSO or PD123319 group (both CD45^+^ inflammatory cells and HSP47^+^ fibroblasts: p<0.01) (Figure 3C).

The mRNA expressions of HSP47, collagen type I alpha 1, collagen type I alpha 2, and collagen type III alpha 1 were also decreased in the valsartan group compared with the DMSO or PD123319 group (HSP47; p<0.05, collage type I alpha 1; p<0.01, collagen type I alpha 2; p<0.05, collagen type III alpha 1; p<0.01) (Figure 3D). These results suggest that the inflammation and fibrosis in cGVHD lacrimal glands were inhibited by the AT1R antagonist (valsartan) administration, accompanied by decreased densities of CD45^+^ inflammatory cells and HSP47^+^ fibroblasts and decreased mRNA expressions of HSP47, collagen type I alpha 1, collagen type I alpha 2, and collagen type III alpha 1.

### Fibrosis of the Lung and Liver in the cGVHD Model Mice is Inhibited by an AT1R Antagonist, but not by an AT2R Antagonist

Because tissue RAS is well known to be involved in fibrotic pathogenesis in the lung and liver, and both organs are major targets for fibrosis in patients with cGVHD, we next asked whether the AT1R antagonist could also decrease the lung and liver fibroses in the cGVHD model mice. First, we examined whether this model would be useful for studying the lung and liver as well as lacrimal gland fibrosis.

**Figure 4:**
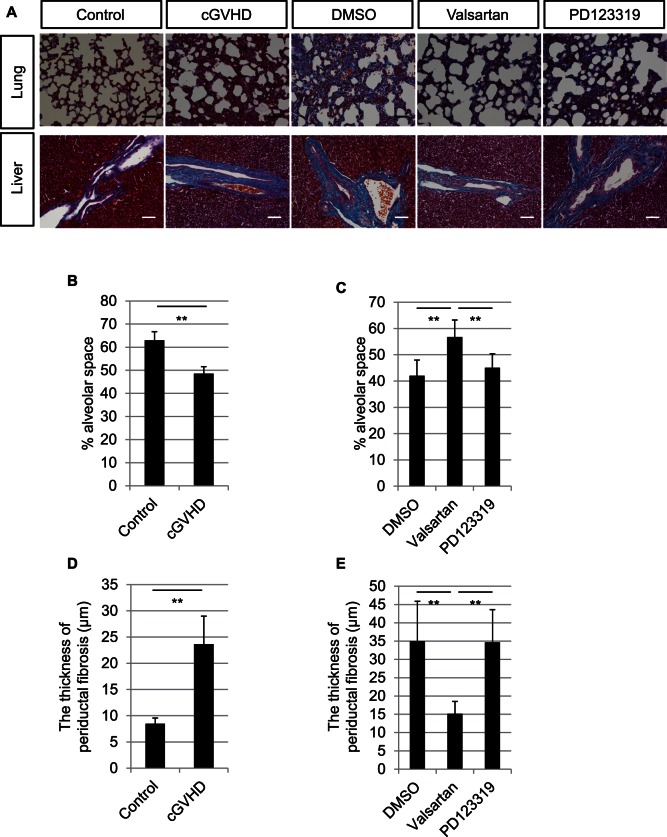
Fibrotic changes in the murine lungs and livers of the DMSO, valsartan, and PD123319 groups. A: Mallory staining of the murine cGVHD lungs showing severe fibrosis of the interstitial space. The interstitial space fibrosis was decreased in the valsartan group. Mallory staining of the murine cGVHD livers showed periductal fibrosis in the bile duct; this fibrosis was decreased in the valsartan group. B: The alveolar space was quantified as the ratio of the alveolar space to the total area in the Mallory staining. The alveolar space was smaller in the cGVHD compared with control lungs (p = 0.0001). C: The alveolar space was largest in the valsartan group (p<0.01). D: The periductal fibrosis was quantified as the mean thickness of blue-stained area around the intrahepatic bile ducts. The fibrosis was thicker in the cGVHD compared with the control livers (p = 0.0003). E: The fibrosis was thinnest in the valsartan group (p<0.01). Magnification, A: X200. Scale bar, A: 50 µm. The results represent the mean ± SD. B, D: Control n = 5, cGVHD n = 5; C, E: DMSO n = 6, valsartan n = 8, PD123319 n = 8. B, D: **p<0.01 by Student's-t test; C, E: **p<0.01 by Bonferroni/Dunn test.

In the lung, the cGVHD model mice showed interstitial space fibrosis with a loss of the normal lacy alveolar pattern (Figure 4A). The alveolar space was smaller in the cGVHD compared with the control lungs (p = 0.0001) (Figure 4B). In the inhibition experiment, valsartan prevented the interstitial space fibrosis (Figure 4A), and the alveolar space was largest in the valsartan group (p<0.01) (Figure 4C). In the liver, the cGVHD model mice showed periductal fibrosis in the bile duct (Figure 4A). The periductal fibrosis was thicker in the cGVHD compared with the control livers (p = 0.0003) (Figure 4D). In the inhibition experiment, valsartan prevented periductal fibrosis in the bile duct (Figure 4A), and the periductal fibrosis was thinnest in the valsartan group (p<0.01) (Figure 4E). These results suggest that fibrosis in cGVHD lung and liver was inhibited by AT1R antagonist (valsartan) administration like that in the lacrimal gland.

## Discussion

Tissue RAS (angiotensin II) has been identified as an important regulator of fibrosis and has been investigated as a potential target of anti-fibrotic drugs [31]. Locally produced angiotensin II is thought to exert its effect by directly inducing NADPH oxidase activity, stimulating transforming growth factor-β1 (TGF-β1) production, and triggering fibroblast proliferation and differentiation into collagen-secreting myofibroblasts [32]–[35]. In this study, we found that fibroses of the lacrimal gland, lung, and liver were increased in cGVHD model mice and ameliorated by an AT1R antagonist.

Our present immunohistochemical analyses showed that the density of fibroblasts increased and that more fibroblasts appeared to express angiotensin II, AT1R, and AT2R in the lacrimal gland of the cGVHD model mice than in that of controls. The fibroblasts in the lacrimal glands of cGVHD model mice were activated and proliferating (i.e., those expressing both HSP47 and Ki67) and their increased number was accompanied by increased depositions of collagen type I and collagen type III. Fibroblasts from several organs are reported to be activated by angiotensin II via paracrine and autocrine actions. For example, angiotensin II accelerates the migration of human dermal fibroblasts [36]. Human lung fibroblasts proliferate and produce collagen in response to AT1R activation [37], [38]. Angiotensin II stimulates the proliferation of cultured renal fibroblasts and increases their expression of mRNAs encoding TGF-β, fibronectin, and collagen type I [39]. Angiotensin II also increases human cardiac fibroblast growth, proto-oncogene expression, and mitogen-activated protein kinase (MAPK) activity, and the expression of mRNAs encoding TGF-β1, fibronectin, and laminin [40]. Pancreatic stellate cells (PaSCs), myofibroblast-like cells found in the exocrine areas of the pancreas, express AT1R and are activated by angiotensin II to promote pancreatic fibrosis [41].

Our findings suggest that fibroblasts may augment the expression of RAS components in the presence of inflammatory events in the cGVHD lacrimal gland microenvironment, resulting in fibroblast activation by locally produced angiotensin II via autocrine and paracrine actions. The number of fibroblasts is then increased as a result of the proliferation and migration of residential and non-residential fibroblasts. The collagen deposition increases, because angiotensin II accelerates the collagen production of the fibroblast or the number of activated fibroblasts is increased. In addition, we found that an AT1R antagonist ameliorated the fibrosis, revealing that these reactions are transmitted mainly by AT1R.

The cGVHD etiology is reported to start with pathogenic donor T cells generated after alloreactivity to host minor histocompatibility antigens or autoantigens occurs [2]. These T cells damage target tissue directly by cytolytic attack, through the secretion of proinflammatory and fibrosing cytokines [2]. In the development of cGVHD, it is reported that macrophages are activated to produce platelet-derived growth factor (PDGF) and TGF-β1, which induce the proliferation and activation of tissue fibroblasts, and that B cells are dysregulated, which leads to the emergence of autoreactive B cells and the production of autoreactive antibodies [42]. All these events contribute to an autoimmune-like systemic syndrome that is associated with fibroproliferative changes. We previously reported that T cell, B cell, and macrophage infiltration and stromal fibroblast activation are found in the periductal areas in the lacrimal gland of cGVHD patients [4]. Electron microscopy showed that lymphocytes infiltrate the periductal areas and destroy the ductal epithelia, and that lymphocytes and macrophages attach to fibroblasts, suggesting that fibroblasts communicate with various inflammatory cells and may play a role in regulating the immune response involved in cGVHD [4]. From these findings, we concluded that both the inflammatory cell infiltration and the fibroblast activation in the periductal area contribute to the fibrotic pathogenesis in the lacrimal glands of cGVHD patients [4].It is reported that RAS can initiate innate and acquired immunity and that T cells and macrophages play pivotal roles in the target-organ damage by responding to angiotensin II in the kidney, heart, and vasculature [43]. In atherosclerosis, angiotensin II promotes the adhesion and infiltration of monocytes/macrophages by up-regulating adhesion molecules and chemokines [44]. Valsartan is reported to reduce macrophage infiltration and the expression of macrophage-derived monocyte chemotactic protein in adipose tissue in mice that results from the inflammatory and metabolic consequences of giving them a high-fat diet [45].

Of the circulating leukocyte subsets, AT1R is reported to be expressed on monocytes, B cells, and T cells [46]. In this study, the mean density of CD45^+^ inflammatory cells was increased in the cGVHD lacrimal gland, and decreased by an AT1R antagonist. It is possible that locally produced angiotensin II accelerated the inflammatory cell infiltration, which was ameliorated by the AT1R antagonist, resulting in the suppression of inflammation and fibrosis in the cGVHD lacrimal gland. Further study is needed to elucidate what subtypes of inflammatory cells are affected by the AT1R antagonist.

Concerning the correlation of lung fibrosis with RAS, hyperoxia increases the collagen and α-smooth muscle actin (α-SMA) expression via RAS components including angiotensinogen and ACE, and angiotensin II expression is also significantly increased by hyperoxic exposure in human lung fibroblasts [47]. AT1R antagonist treatment attenuates the hyperoxia-induced lung fibrosis and decreases the hyperoxia-induced expression of extracellular signal-regulated protein kinase and α-SMA in hyperoxia-exposed newborn rats [22]. Furthermore, the binding of angiotensin II to AT1R induces the apoptosis of alveolar epithelial cells, which is important for the pathogenesis of lung fibrosis [48]. It is possible that tissue RAS was up-regulated in the cGVHD lung, which induced the apoptosis of alveolar epithelial cells and fibroblast activation. AT1R antagonist administration increased the alveolar space, possibly because the interstitial space fibrosis resulting from the apoptosis of alveolar epithelial cells, fibroblast proliferation, and intraseptal collagen deposition was suppressed.

In the liver fibrosis following liver injury, ACE, and AT1R are markedly increased and are localized to areas of active fibrogenesis [18]. ACE and AT1R are reported to be highly expressed in activated human HSCs (hepatic stellate cells), which play a key role in liver fibrosis [49]. An AT1R antagonist attenuates the fibrosis development in pig serum-induced liver fibrosis in rats by blocking the expression of TGF-β mRNA, which is increased by locally produced angiotensin II in the activated HSCs [21]. It is possible that tissue RAS was up-regulated in the fibrotic area in the cGVHD liver with HSC activation, and that the AT1R antagonist suppressed the hepatic fibrosis by suppressing the HSC activation. Further study is needed to investigate how cGVHD affects the expression of RAS components and how AT1R antagonists suppress fibrosis. In addition, the impact of the AT1R antagonist on the kinetics of the fibrosis must be determined at different doses and times after BMT.

In the present study, we used HSP47 to assess the degree of lacrimal gland fibrosis; we examined the number of HSP47^+^ fibroblasts and the mRNA expression of HSP47. HSP47 is a stress-inducible 47-kDa collagen-binding glycoprotein that resides in the endoplasmic reticulum of collagen-producing cells, and is involved in the post-translational modifications and triple-helix structure of procollagen molecules [50]. Increased HSP47 expression with excessive collagen accumulation is consistently observed in various human and experimental fibrotic diseases [51], and the upregulation of HSP47 is commonly observed during the collagenization of an involved organ, regardless of the primary disease [51]. Regarding the correlation between HSP47 and the fibrosis of cGVHD, we previously reported that the lacrimal glands of patients with cGVHD show a marked increase in HSP47 expression in fibroblasts around the medium-sized ducts in interlobular areas [29]. Considering that the distribution of collagen III immunoreactivity is similar to the HSP47 distribution, we proposed that the increased expression of HSP47 may promote excessive collagen assembly in and around the periductal area. In addition, we reported that HSP47-expressing fibroblasts in the periductal area show almost no α-SMA staining in serial sections of the cGVHD lacrimal gland, and that primary fibroblast cultures derived from the cGVHD lacrimal gland show higher HSP47 mRNA expression, but lower levels of α-SMA mRNA and protein expression, and fewer α-SMA-positive cells than do fibroblasts isolated from Sjögren's syndrome patients (used as a control for cGVHD in the article). Therefore, we concluded that fibroblasts have relatively low myofibroblast transformation, suggesting that HSP47-positive fibroblasts rather than α-SMA-positive myofibroblasts are the main matrix-regulating cells and that low α-SMA expression appears to be unique to the lacrimal glands of patients with cGVHD [29].

In the present study, we examined the HSP47 and collagen mRNA expressions in the whole lacrimal gland in cGVHD model mice and revealed that HSP47 mRNA expression was significantly enhanced in the cGVHD lacrimal glands, and this enhancement was accompanied by increases in the mRNA expressions for collagen type I alpha 1, collagen type I alpha 2, and collagen type III alpha 1. Our subsequent experiments showed that the administration of an AT1R antagonist prevented the development and progression of lacrimal gland fibrosis. We propose two mechanisms. First, the AT1R antagonist might ameliorate fibrosis by decreasing the expression of HSP47 and collagen in the cGVHD lacrimal gland. Second, the AT1R antagonist might suppress the number of fibroblasts expressing high levels of HSP47 and collagen, resulting in decreased expressions of HSP47 and collagen in the whole lacrimal gland. Further studies are necessary to elucidate the precise mechanism by which tissue RAS accelerates and AT1R antagonist suppresses the expression of HSP47 and collagen. In addition, these results suggest that the mRNA expression of HSP47 can be used as a fibrotic index that reflects the degree of fibrosis in the lacrimal gland of the cGVHD model mouse.

In summary, we found that tissue RAS played a pivotal role in the fibrosis of the cGVHD lacrimal gland and that an AT1R antagonist had a therapeutic effect on fibrosis of the lacrimal gland, lung, and liver. Because AT1R antagonists are widely used in clinical practice without serious side effects, these drugs may provide an effective new strategy for anti-fibrotic therapy in cGVHD.

## Supporting Information

Figure S1 **FISH for Y-chromosomes using spleen cells from female recipient mice that had undergone male whole bone marrow transplantation.** Cells with a Y-FISH signal in the nucleus were regarded as donor-derived male cells (white arrows and magnified in inset). Three weeks after bone marrow transplantation, about 40% of the peripheral blood cells were donor-derived. The detection sensitivity of the Y-FISH signal was found to be 45% in the positive control. Therefore, the engraftment efficiency was estimated to be about 88.8%. Magnification: X400, Scale bar: 20 µm.
(EPS)

Figure S2 **Immunofluorescence double staining with HSP47 and Ki-67, collagen type I, or collagen type III in control and cGVHD murine lacrimal glands.** Fibroblasts in the cGVHD lacrimal gland co-expressing HSP47 and Ki-67 (arrowheads) were scarcely detected in the control. Immunostainings of collagen type I and collagen type III were stronger in the interstitium, and more fibroblasts co-expressed HSP47 and collagen type I or collagen type III in the cGVHD than in the control lacrimal glands. Magnification: X400, Scale bar: 20 µm.
(EPS)

Materials and Methods S1 Procedure for spleen cell cytospin: Spleen cells were obtained from female transplant recipient mice that had received male whole bone marrow transplantation (BMT). A 2-ml sample containing 5×10^5^ spleen cells was added to the chamber of a silane-coated glass slide. After centrifugation at 2,000 rpm for 5 min, the slides were removed from the cytospin chamber, and the cells were fixed in 10% neutral buffered formalin for 10 minutes at 4°C until use. Fluorescein in situ hybridization (FISH) for Y-chromosomes: Cytospun spleen cells were subjected to Y-chromosome FISH using a DNA probe for the mouse Y-chromosome (Cambio, Cambridge, U.K.) and re-staining with TO-PRO-3 (Invitrogen/Molecular Probes). The slides were observed under a confocal microscope (LSM700-ZEN 2009, Carl Zeiss Microlmaging GmbH, Jena, Germany).
(DOCX)
