# Supplementary material for: Angiotensin II Type 1 Receptor Antagonist Attenuates Lacrimal Gland, Lung, and Liver Fibrosis in a Murine Model of Chronic Graft-Versus-Host Disease
Source: PLoS One. 2013 Jun 6;8(6):e64724. doi: 10.1371/journal.pone.0064724 (PMC3675140; doi:10.1371/journal.pone.0064724)
Supplement: Materials and Methods S1 — Procedure for spleen cell cytospin: Spleen cells were obtained from female transplant recipient mice that had received male whole bone marrow transplantation (BMT). A 2-ml sample containing 5×10^5^ spleen cells was added to the chamber of a silane-coated glass slide. After centrifugation at 2,000 rpm for 5 min, the slides were removed from the cytospin chamber, and the cells were fixed in 10% neutral buffered formalin for 10 minutes at 4°C until use. Fluorescein in situ hybridization (FISH) for Y-chromosomes: Cytospun spleen cells were subjected to Y-chromosome FISH using a DNA probe for the mouse Y-chromosome (Cambio, Cambridge, U.K.) and re-staining with TO-PRO-3 (Invitrogen/Molecular Probes). The slides were observed under a confocal microscope (LSM700-ZEN 2009, Carl Zeiss Microlmaging GmbH, Jena, Germany). (DOCX) [file pone.0064724.s003.docx]

**Materials and methods S1**

**Procedure for spleen cell cytospin**

Spleen cells were obtained from female transplant recipient mice that had received male whole bone marrow transplantation (BMT). A 2-ml sample containing 5x10^5^ spleen cells was added to the chamber of a silane-coated glass slide. After centrifugation at 2,000 rpm for 5 min, the slides were removed from the cytospin chamber, and the cells were fixed in 10% neutral buffered formalin for 10 minutes at 4°C until use.

**Fluorescein in situ hybridization (FISH) for Y-chromosomes**

Cytospun spleen cells were subjected to Y-chromosome FISH using a DNA probe for the mouse Y-chromosome (Cambio, Cambridge, U.K.) and re-staining with TO-PRO-3 (Invitrogen/Molecular Probes). The slides were observed under a confocal microscope (LSM700-ZEN 2009, Carl Zeiss Microlmaging GmbH, Jena, Germany).
